# Ultrathin Descemet Stripping Automated Endothelial Keratoplasty (UT-DSAEK) versus Descemet Membrane Endothelial Keratoplasty (DMEK)—a systematic review and meta-analysis

**DOI:** 10.1038/s41433-023-02467-2

**Published:** 2023-03-18

**Authors:** Daire J. Hurley, Patrick Murtagh, Marc Guerin

**Affiliations:** Department of Ophthalmology, Mater Misericordiae University Hospital, Eccles Street, Dublin 7, Ireland

**Keywords:** Eye diseases, Outcomes research

## Abstract

**Background/Objectives:**

Endothelial keratoplasty (EK) is a commonly performed transplant procedure used in the treatment of corneal endothelial dysfunction. The aim of this systematic review and meta-analysis is to evaluate the differences in visual acuity outcomes, endothelial cell density (ECD) and complications between two forms of EK, ultrathin Descemet stripping automated endothelial keratoplasty (UT-DSAEK) and Descemet membrane endothelial keratoplasty (DMEK).

**Methods:**

A literature search of MEDLINE, Embase and Cochrane Library was conducted to identify studies reporting comparative results of UT-DSAEK versus DMEK. The Preferred Reporting Items for Systematic Reviews and Meta-Analyses (PRISMA) statement was used for search strategy. Of 141 titles, 7 studies met the inclusion criteria; best corrected visual acuity (BCVA) (LogMAR), ECD (cells/mm^2^), and complications were compared, with all statistical analysis performed using Review Manager.

**Results:**

A total of 362 eyes were included for analysis. DMEK resulted in significantly better BCVA at 3 months (0.14 vs 0.22, *p* = 0.003), 6 months (0.08 vs 0.18, *p* = 0.005) and 1 year post-op (0.07 vs 0.14, *p* = 0.0005). UT-DSAEK resulted in significantly lower total complications (25.2% vs 57.3%, *p* = 0.0001) and rates of re-bubbling (11.0% vs 33.7%, *p* = 0.004). No differences were found in ECD between the two procedures (1541 vs 1605, *p* = 0.77).

**Conclusions:**

DMEK results in superior visual acuity rates with quicker recovery. However, UT-DSAEK has a more favourable complication profile, particularly regarding lower rates of re-bubbling. Both are valuable options in the treatment of corneal endothelial disease and choice of procedure may depend on surgical expertise.

## Introduction

Corneal transplantation is the most performed type of allogeneic transplantation worldwide with approximately 185,000 keratoplasties being undertaken place annually [1]. Since the first transplantation in 1905, the procedure has seen advances from full thickness transplantation, known as penetrating keratoplasty (PK), to the selective replacement of damaged layers, known as lamellar keratoplasty. Most progress has been seen in endothelial keratoplasty (EK), a procedure in which the diseased corneal endothelium is replaced. The corneal endothelium plays an essential role in maintaining corneal hydration and clarity through the endothelial Na + /K + ATPase pump and tight junctions between endothelial cells that tightly regulate the transport of fluid into the stroma [2]. The average adult cornea has an endothelial cell density (ECD) of approximately 2500–3000 cells/mm^2^, with a reduction of approximately 0.6% per year [3]. When endothelial cell density falls below 500–800 cells/mm^2^, decompensation may occur leading to corneal oedema and necessitating corneal transplantation.

EK is the gold standard in treating corneal endothelial disease as it offers quicker recovery, improved visual acuity and fewer complications compared to PK. It is most commonly indicated for Fuchs’ endothelial dystrophy (47.1%), followed by pseudophakic bullous keratopathy (17.5%) [4]. Within the scope of EK, there has been a shift towards the use of thinner grafts. Descemet’s stripping automated endothelial keratoplasty (DSAEK) involves the transplantation of the posterior stroma, Descemet membrane and the endothelium. By comparison, Descemet membrane endothelial keratoplasty (DMEK) involves the removal and transplantation of just Descemet’s membrane and the endothelium. DMEK has been shown to further improve visual acuity and reduce rates of graft rejection [5]. However, DMEK is a technically challenging procedure with higher rates of operative complications due to the thinness and fragility of the graft [6].

Ultrathin-DSAEK (UT-DSAEK) is a relatively new form of EK that may bridge the gap between DSAEK’s less challenging use and DMEK’s superior results. UT-DSAEK is commonly agreed to comprise donor grafts of approximately <110–100 μm thick, smaller than the traditional DSAEK’s 150 μm and larger than DMEK’s average 10–15 μm thickness [7, 8]. This is achieved through a single or double microkeratome pass, the first pass debulking the donor tissue and the second pass refining it to the optimal thickness [9]. UT-DSAEK was shown to have superior visual acuity outcomes compared to the traditional DSAEK procedure in a large case series [10] and randomised controlled trial [11] without increasing the risk of graft detachment.

To date, there have been a small number of randomized controlled trials comparing UT-DSAEK and DMEK but no meta-analyses performed on the topic. The aim of this study is to compare best corrected visual acuity (BCVA) outcomes, ECD and complication rates between the two procedures.

## Methods

### Study selection

A literature search based on the Preferred Reporting Items for Systematic Reviews and Meta-Analyses (PRISMA) guidelines was performed by two independent reviewers (DJH and PM) with the senior author (MG) arbitrating on any disagreement. The title and abstract were reviewed for all search results and potentially eligible studies received a full-text review. Finally, the reference lists of the included studies and literature reviews found in the initial search were manually screened for additional articles meeting the inclusion criteria.

### Search strategy

The following search terms were used in the PubMed/MEDLINE, Embase and Cochrane Library databases on the 20^th^ September 2022 with the search algorithm: “(Ultrathin Descemet Stripping Automated Endothelial Keratoplasty OR UT-DSAEK OR Ultrathin Descemet Stripping Endothelial Keratoplasty UT-DSEK) AND (Descemet Membrane Endothelial Keratoplasty OR DMEK)”. No time limit was given to publication date.

### Eligibility criteria

The inclusion criteria were as follows: [1] clinical studies comparing UT-DSAEK and DMEK, including randomised controlled trials and cohort studies, with at least 1 year follow-up. UT-DSAEK was defined as a graft thickness of 50–105 μm in keeping with the literature; [2] publication in a peer-reviewed journal; [3] publication in English; and [4] studies for which the full text was available. The exclusion criteria were [1] review studies; [2] publication not in English; and [3] abstract-only publications.

### Data extraction and analysis

Study characteristics were collected by a blinded reviewer using a predetermined data sheet. These characteristics included: [1] the study design, [2] level of evidence (LOE), [3] methodological quality of evidence (MQOE), [4] population, [5] clinical outcome measures and [6] follow-up time points. Clinical outcomes of interest included; [1] BCVA, [2] ECD, and [3] complications.

The MQOE was assessed by two independent reviewers. This was assessed using the Newcastle-Ottawa scale for cohort studies [12]. On this 9-point scale, studies receiving 7 to 9 points, 5 to 6 points, 4 points, and 0 to 3 points were graded as very good, good, satisfactory, and unsatisfactory, respectively. Randomised controlled trials were assessed using the Cochrane Collaboration risk of bias tool [13]. Studies were considered as having a low risk of bias when every single item was scored as ‘low risk’. Studies were considered as moderate risk of bias when ‘high risk’ or ‘unclear risk” on one or two items of bias was scored. Studies were considered as high risk of bias when more than two ‘high risk’ or ‘unclear risk’ were scored. The GRADE Working Group grades of evidence were then used to assess quality of evidence.

### Statistical analysis

All statistical analysis was performed using Review Manager (RevMan for Macintosh, version 5.3 [2014]; The Nordic Cochrane Centre, The Cochrane Collaboration, Copenhagen, Denmark). Random-effects models were used. Results were presented as the risk ratio (RR) for dichotomous outcomes with the 95% confidence interval (CI). Heterogeneity between studies was quantified using the *I*^2^ statistic [13]. An *I*^2^ value of less than 25% was chosen to represent low heterogeneity; greater than 75%, high heterogeneity. *P* < 0.05 was considered statistically significant.

## Results

### Literature search

The initial literature search identified a total of 141 studies. After removal of duplicates, the articles were screened for the inclusion and exclusion criteria; 68 unique studies were evaluated and 27 full texts were assessed for eligibility. Seven studies with 362 eyes (322 patients) were included in this review [14–20]. Rose-Nussbaumer et al. (2021) is a 2 year follow-up of the same cohort as Chamberlain et al. (2019) and this has been included for analysis as one paper, the DETECT (Descemet Endothelial Thickness Comparison Trial) study, to avoid duplication. The PRISMA (Preferred Reporting Items for Systematic Reviews and Meta-Analyses) flowchart can be found in the appendix.

### Study characteristics and patient demographics

There were three randomised controlled studies (Level I evidence) and four retrospective cohort studies (Level III evidence). The mean MQOE score of the studies was 7.6. The seven studies compared 189 eyes treated with UT-DSAEK versus 173 treated with DMEK with a minimum of one year follow-up. Most of the patients (65.4%) were female; the average age was 71.6 years and the most common indication was Fuchs’ endothelial dystrophy (91.7%). The study characteristics are shown in Table 1. The baseline visual acuity and endothelial cell density measures of patients were similar between the cohorts. The average graft thickness in the UT-DSAEK group was 70.4 μm ± 4.5 μm. A triple procedure entailed EK, either UT-DSAEK or DMEK, with cataract removal and intra-ocular lens insertion. This was performed in 77 UT-DSAEK eyes (40.7%) and 86 DMEK eyes (49.7%). All patients in all studies were pseudophakic post-operatively. The pre-operative and operative data can be found in Table 2.

**Table 1:** Study characteristics and patient demographics.

Study	LOE	Design	MQOE Score	Follow-Up (mo)	No. of Eyes (No. of Patients)		Age (yrs.)		Female (%)		FED (%)	
					UT-DSAEK	DMEK	UT-DSAEK	DMEK	UT-DSAEK	DMEK	UT-DSAEK	DMEK
DETECT study, 2021	I	RCT	Low	24	25 (N.S.)^a^	25 (N.S.)^a^	68 ± 11	68 ± 5	64	52	96	96
Dunker, 2020	I	RCT	Low	12	25(25)	29 (29)	71 ± 7	72 ± 7	N/A	N/A	100	100
Matsou, 2021	I	RCT	Low	12	28 (28)	28 (28)	72 ± 10.32	73 ± 7.5	50	68	100	100
Mencucci, 2020	III	RCS	8	12	18 (18)^b^	18 (18)^b^	73.5 ± 7.9	73.5 ± 7.9	88.9	88.9	100	100
Romano, 2020	III	RCS	6	12	31 (31)	25 (25)	69.3 ± 13	77.6 ± 9.7	N/A	N/A	51.6	72
Torras-Sanvicens, 2021	III	RCS	7	30.5	10 (10)^b^	10 (10)^b^	75.4 ± 6.7	75.4 ± 6.7	60	60	100	100
Tourabaly, 2019	III	RCS	7	32	52 (52)	38 (38)	72 ± 7	69 ± 8	N/A	N/A	90.4	97.4

*FED* Fuchs endothelial dystrophy, *LOE* level of evidence, *mo* months, *N.S.* not specified, *RCS* retrospective cohort study, *RCT* randomized controlled trial.

^a^50 eyes in total from 38 patients

^b^Fellow eye comparison study.

**Table 2:** Pre-operative and operative data.

Study	Pre-Operative BCVA (logMAR)		Pre-Operative ECD (cells/mm^2^)		Pre-Operative CCT (μm)		Triple Procedure (%)		Graft Thickness (μm)		Gas Tamponade (% using Air only)	
	UT-DSAEK	DMEK	UT-DSAEK	DMEK	UT-DSAEK	DMEK	UT-DSAEK	DMEK	UT-DSAEK	DMEK	UT-DSAEK	DMEK
DETECT study, 2021	0.27 ± 0.21	0.34 ± 0.29	2796 ± 238	2771 ± 150	610 ± 44	608 ± 52	68	72	73 ± 12	N/A	N.S.	N.S.
Dunker, 2020	0.31 ± 0.13	0.37 ± 0.18	2633 ± 158	2679 ± 157	N/A	N/A	0	0	101 ± 25	N/A	100	41.4
Matsou, 2021	0.38 ± 0.23	0.38 ± 0.15	2632 ± 163	2682 ± 205	N/A	N/A	71	86	63 ± 12.9	N/A	100	0
Mencucci, 2020	0.6 ± 0.29	0.51 ± 0.11	2700 ± 59	2625 ± 125	618 ± 39	629 ± 39	0	0	80.3 ± 20.5	N/A	100	100
Romano, 2020	1.09 ± 0.7	0.84 ± 0.58	2562 ± 111	2560 ± 10	N/A	N/A	35.5	60	75.3 ± 15.4	N/A	N.S.	N.S.
Torras-Sanvicens, 2021	0.49 ± 0.22	0.43 ± 0.17	2520 ± 257	2670 ± 195	671 ± 61	656 ± 39	20	0	91.1 ± 25.2	N/A	100	100
Tourabaly, 2019	0.84 ± 0.38	0.48 ± 0.31	N/A	N/A	661 ± 77	662 ± 58	51.9	76.3	78 ± 14	N/A	0	0

*BCVA* best corrected visual acuity, *CCT* central corneal thickness, *ECD* endothelial cell density, *N/A* not applicable, *N.S.* not specified.

### Clinical outcomes

#### Best corrected visual acuity (BCVA)

**Fig. 1:**
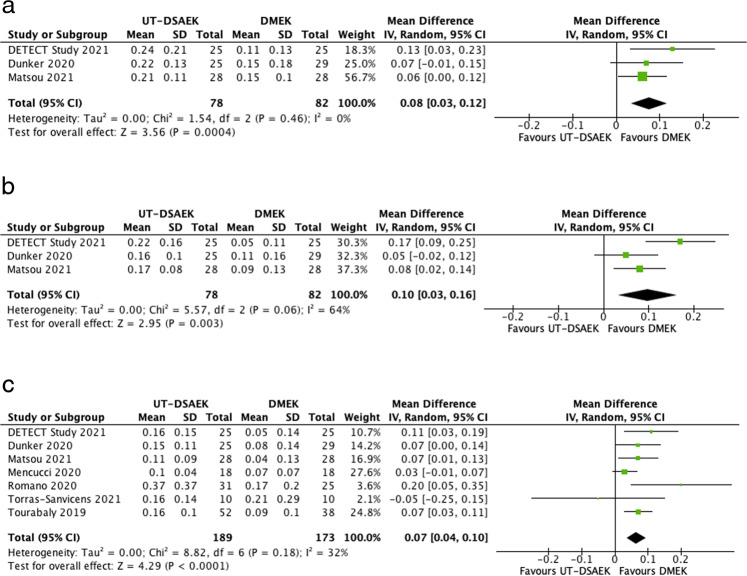
Forest plot of best corrected visual acuity (LogMAR). **a** Forest plot of Best Corrected Visual Acuity (LogMAR) at 3 months. **b** Forest plot of Best Corrected Visual Acuity (LogMAR) at 6 months (**c**) Forest plot of Best Corrected Visual Acuity (LogMAR) after 1 year.

Three studies reported BCVA at 3 months and 6 months follow-up, with 78 eyes in the UT-DSAEK cohort and 82 in the DMEK cohort. A statistically significant difference was observed in favour of DMEK at 3 months (0.14 vs 0.22, CI, 0.03–0.12; *I*^2^ = 0%, *p* < 0.001) and at 6 months (0.08 vs 0.18, CI, 0.03–0.16; *I*^2^ = X%, *p* = 0.003). All seven studies reported BCVA after 1 year follow-up and DMEK remains the superior option at this point (0.07 vs 0.14, CI, 0.04–0.10; *I*^2^ = 32%, *p* < 0.001). The forest plots for BCVA are shown in Fig. 1.

#### Endothelial cell density (ECD)

**Fig. 2:**
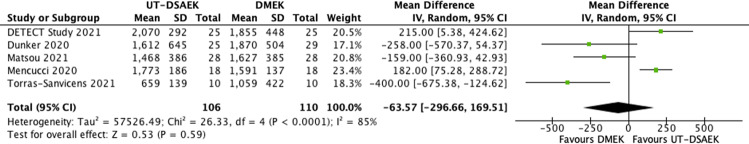
Forest plot of endothelial cell density (cells/mm2) after 1 year. CI Confidence Interval, SD Standard Deviation.

ECD at one year was reported in five studies, with 106 eyes in the UT-DSAEK group and 110 in the DMEK group. Mean ECD was 1541 cells/mm^2^ after one year following UT-DSAEK and 1605 cells/mm^2^ following DMEK. No difference was found between the two procedures (CI, −296.7–169.5; *I*^2^ = 85%, *p* = 0.59). The forest plot for ECD is shown in Fig. 2.

#### Complications

**Fig. 3:**
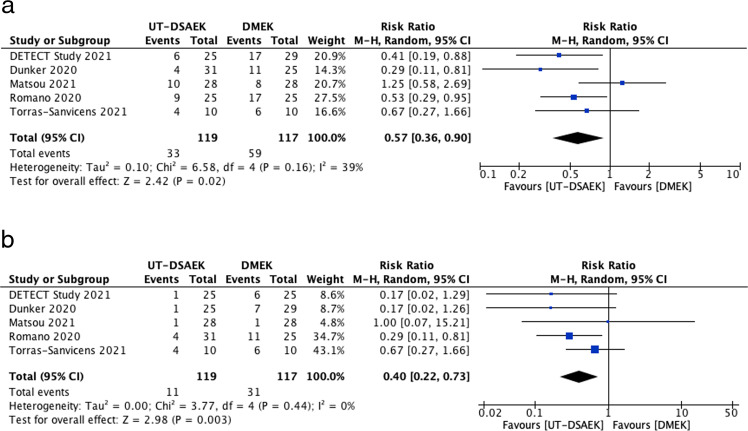
Forest plot of complications. **a** Forest plot of Total Complications. **b** Forest plot of rates of Re-Bubbling.

Total complications were reported in five studies, with 119 eyes in the UT-DSAEK cohort and 117 in the DMEK cohort. Overall, 33 eyes (27.7%) had complications following UT-DSAEK, whereas 59 (50.4%) of eyes in the DMEK cohort had a complication. UT-DSAEK was associated with significantly lower total complications (RR, 0.57; CI, 0.36–0.9; *I*^2^ = 39%; *p* = 0.02). The most common complication was the need for re-bubbling, occurring in 11 eyes (9.2%) in the UT-DSAEK cohort and 31 eyes (26.5%) in the DMEK group (RR, 0.4; CI, 0.22–0.73; *I*^2^ = 0%, *p* = 0.003). This was followed by glaucoma or a raised intra-ocular pressure (IOP), occurring in 15 eyes (12.6%) post-UT-DSAEK and 14 eyes (12.0%) post-DMEK. The full list of complications can be found in the appendix. The forest plots for complications are shown in Fig. 3.

## Discussion

DMEK results in visual acuity outcomes of close to one-line superiority over UT-DSAEK at all post-operative visits. This difference is most pronounced in the initial 3 months post-operatively, highlighting its faster visual rehabilitation, but it remains significantly better at 6 months and 1 year follow-up with high quality of evidence. This is likely due to the improved restoration of anatomy in the DMEK procedure because of the use of a thinner graft with no lamellae and lack of stroma-to-stroma graft interface. Thinner grafts have been shown to induce fewer higher order aberrations and less hyperopic shift which may explain DMEK’s visual acuity superiority of UT-DSAEK [21–23]. DMEK procedures also utilise smaller incisions, 2.2–3.5 mm for DMEK versus 3.5–4.5 mm for UT-DSAEK, which results in less surgically induced astigmatism [24]. Both Mencucci et al. and Torras-Sanvicens et al. performed retrospective fellow eye comparisons of patients who underwent UT-DSAEK in one eye and DMEK in the other [16, 20]. Although, they found similar BCVA at one year follow-up, DMEK performed better in terms of contrast sensitivity, posterior corneal aberrations and overall patient satisfaction.

Some discordance in visual acuity outcomes exists between our randomised controlled trials [8, 14, 15, 17]. All three studies used strict eligibility criteria, only analysing patients with endothelial disease, primarily Fuchs’ endothelial dystrophy, in the absence of other vision-limiting pathologies. Dunker et al. found no significant differences in visual acuity between the two procedures as early as 3 months post-operatively and up to 1 year [14]. They assessed outcomes in pre-operatively pseudophakic eyes to isolate the effect of keratoplasty alone on visual outcome. Although this doesn’t reflect the heterogeneity of the patient population attending for endothelial keratoplasty, it does suggest that the outcomes analysed are primarily attributable to the procedure alone. They did however report that a higher percentage of patients attained 0.1 LogMAR in the DMEK arm (66% of 29 eyes) compared to the UT-DSAEK arm (33% of 25 eyes). The DETECT and Matsou studies showed superior visual acuity in the DMEK group. 70% of patients in the DETECT cohort had a triple procedure. This, however, was done in equal proportion between both procedures, 68% in the UT-DSAEK group and 72% in the DMEK group. Although this may affect pre-operative BCVA, all patients analysed across all studies were pseudophakic at the end of their keratoplasty allowing comparable analysis of post-operative BCVA at one year.

The variability in outcomes between the studies may also relate to an inherent limitation in UT-DSAEK that is the lack of graft regularity. Graft regularity is an important parameter in the quality of an UT-DSAEK graft as and remains difficult to standardize, even with eye bank-prepared tissues [21]. Despite this, the DETECT study and Matsou et al. found no difference in patient-reported functional vision as assessed by vision related quality of life [15, 25]. This is in line with Matsou and Dunker’s findings of no significant difference in mean spherical equivalent, posterior and anterior corneal astigmatism between the two cohorts at any time point [14, 15].

ECD is a major factor in long-term graft survival [26]. The Cornea Preservation Time Study evaluated factors influencing graft success and they found higher success in cases of Fuchs’ endothelial dystrophy, cases without any intra-operative complications and in which the donor did not have diabetes [27]. In our analysis, we found ECD was comparable between the procedures. This is in line with multiple RCTs looking at ECD between DMEK and traditional DSAEK which have found no significant difference in the early post-operative period [6, 28, 29].

The rate of total complications is markedly higher after DMEK than after UT-DSAEK. The most reported complication in each cohort was graft detachment requiring re-bubbling (anterior chamber tamponade re-injections). This is a significant concern following DMEK [18]. Romano et al. reported that re-bubbling was also higher in eyes undergoing triple procedure, occurring in 3/11 eyes having UT-DSAEK-triple and 8/15 undergoing DMEK-triple. However, contradictory results in other case series have shown that combining cataract procedures with DSAEK [30] or DMEK [31] has no impact on rates of detachment. The reported rates of re-bubbling following DMEK vary widely, ranging from 2% to 82% but on average are considered to occur in just under one-third of patients [32, 33]. Repeated re-bubblings are associated with endothelial cell loss, raised intraocular pressure and graft rejections [6]. Re-bubbling also poses additional logistic issues. It may have to be performed in theatre where access can often be an issue and may take precedence over other elective ophthalmic procedures. However, DMEK re-bubbling may be more frequently performed in clinic, compared to UT-DSAEK which often requires management in theatre owing to the higher pressure required for successful tamponade. Graft detachment rates may be reduced with the use of pre-stamped DMEK tissue [34]. Additionally, studies have shown that SF6 gas anterior chamber tamponade may further reduce the risk of detachment to approximately 12% by facilitating adhesion at the graft-host interface [35–37]. The DETECT study and Dunker et al. noted that the use of SF6 made no statistical difference to rates of re-bubbling, although they weren’t powered to specifically analyse this [8, 14, 17]. Studies have also shown that the use of SF6 tamponade does not affect long-term BCVA or ECD [35–37].

Raised IOP was the second most reported complication in each cohort. It was not clearly stated what percentage of patients went on to develop secondary glaucoma. This raised IOP is likely a result of post-operative steroid use but may also develop from air bubble-related pupillary block [38]. Prophylactic peripheral iridotomy may be performed pre- or intra-operatively to reduce the risk of pupillary block in either procedure [39].

Rates of graft failure (2 in DMEK and 1 in UT-DSAEK) and rejection were very low (1 in DMEK and 2 in UT-DSAEK). However, longer follow-up is required to comment on the risk of risk of allograft rejection and graft survival. As less tissue is transplanted in DMEK, there should be a reduced risk of allograft rejection and less reliance on topical steroids. A multi-centre study of 431 patients who underwent DMEK found a 3.7% (16 eyes) graft rejection rate [40]. This is significantly lower than the rejection rates commonly reported following traditional DSAEK (9%) [41]. UT-DSAEK may have lower rates of rejection compared to its predecessor, DSAEK, with its thinner graft and a large prospective series has shown similar rates of immunologic rejection to DMEK [10].

An important consideration is the use of eye bank versus surgeon prepared endothelial grafts. Romano et al. looked at DMEK graft preparation and found significantly higher adhesion and lower re-bubbling rates with surgeon prepared DMEK grafts compared to eye bank prepared tissue [18]. Similar results were seen in a study examining re-bubbling rates in DSAEK procedures, with fewer detachments occurring in the surgeon prepared group [42]. The use of surgeon or local technician prepared endothelial grafts, both DMEK and traditional DSAEK, have also been shown to significantly reduce surgical expenses in Canada and the United Kingdom [42, 43]. These studies did not specifically analyse surgeon prepared UT-DSAEK grafts. However, both single and double-pass microkeratome techniques for performing have been well described in the literature with reproducible results [44–46]. Techniques to improve graft preparation consistency include controlling artificial anterior chamber pressure and drying the corneal surface, described by Romano et al., and stromal swelling with balanced salt solution prior to microkeratome pass, detailed by Farbman et al. Single and double pass techniques for UT-DSAEK graft preparation have been shown to have comparable thickness, ECD and BCVA [47].

In 2015, according to the Eye Bank Association of America, DMEK comprised 15% of all EK procedures in the United States, whereas DSAEK accounted for over 50%. In 2021, their usage was almost equal (DMEK: 14,128 vs DSAEK: 15,935) [48]. DMEK’s widespread adoption may have initially been limited by its highly technical nature, at every stage from donor tissue preparation to placement to post-operative graft attachment. Owing to the fragility of the tissue, graft folds occur in approximately 1.9% which may cause optical aberrations [40]. Many studies have shown a steep learning curve for DMEK with an inverse relationship between surgeon experience and rates of graft detachment [49–51]. Dapena et al. examined the outcomes in a series of consecutive cases performed by a single surgeon and found re-bubbling was required in 20% of the first 45 cases, 13.3% in the middle 45 cases and 4.4% in the final 45 cases [49]. DMEK is also a more challenging procedure in patients with complex anatomy, such as a very shallow or deep anterior chamber or a history of prior intra-ocular surgery or trauma [52]. Visual outcomes and endothelial cell loss following DMEK remain stable however once the surgeon has completed a minimum of 25 cases [50, 51].

### Limitations

This study has several limitations and potential biases, including the limitations of the included studies themselves. 4 of the 7 studies were retrospective thus potentiating selection and performance bias. It was not possible to adjust for age, sex or diagnosis. However, there was no significant difference in these demographic variables between the 2 groups. Additionally, the lack of a standardised graft thickness in UT-DSAEK remains a challenge in reporting its outcomes accurately. The overall heterogeneity was relatively low for visual acuity outcomes, at 3 months and 1 year, and complication measures, indicating a degree of consistency between these results in the studies. Nevertheless, high heterogeneity was seen in visual acuity outcomes at 6 months and ECD results, as indicated by the discrepancies in values between the studies.

## Conclusion

In summary, visual outcomes were superior following DMEK with quicker recovery and better overall visual acuity. Conversely, UT-DSAEK is associated with fewer complications, particularly graft detachments requiring re-bubbling. Both procedures remain valuable options for a cornea specialist with prudent decision making given to the right operation for the right patient. Further large multicentre randomised controlled trials are required to further clarify differences between the two procedures, particularly in terms of endothelial cell loss, graft rejection and overall graft survival.

### Summary

#### What was known before

- Patients who undergo DMEK have better visual acuity outcomes and reduced rates of graft rejection compared to those with traditional DSAEK. Ultrathin DSAEK (UT-DSAEK) utilises a donor graft of approximately 50–100 um thickness, smaller than the traditional DSAEK.

#### What this study adds

- DMEK results in superior visual acuity rates with quicker recovery compared to UT-DSAEK. UT-DSAEK has a more favourable complication profile, particularly regarding lower rates of re-bubbling. Endothelial cell density is comparable between the two procedures after 1 year.

### Supplementary information

Appendix
