# Supplementary material for: Ultrathin Descemet Stripping Automated Endothelial Keratoplasty (UT-DSAEK) versus Descemet Membrane Endothelial Keratoplasty (DMEK)—a systematic review and meta-analysis
Source: Eye (Lond). 2023 Mar 18;37(14):3026–32. doi: 10.1038/s41433-023-02467-2 (PMC10516931; doi:10.1038/s41433-023-02467-2)
Supplement: Supplementary file 1 — Appendix [file 41433_2023_2467_MOESM1_ESM.docx]

**Appendix 1.** PRISMA (Preferred Reporting Items for Systematic Reviews and Meta-Analyses) Study Selection Flow Diagram

**
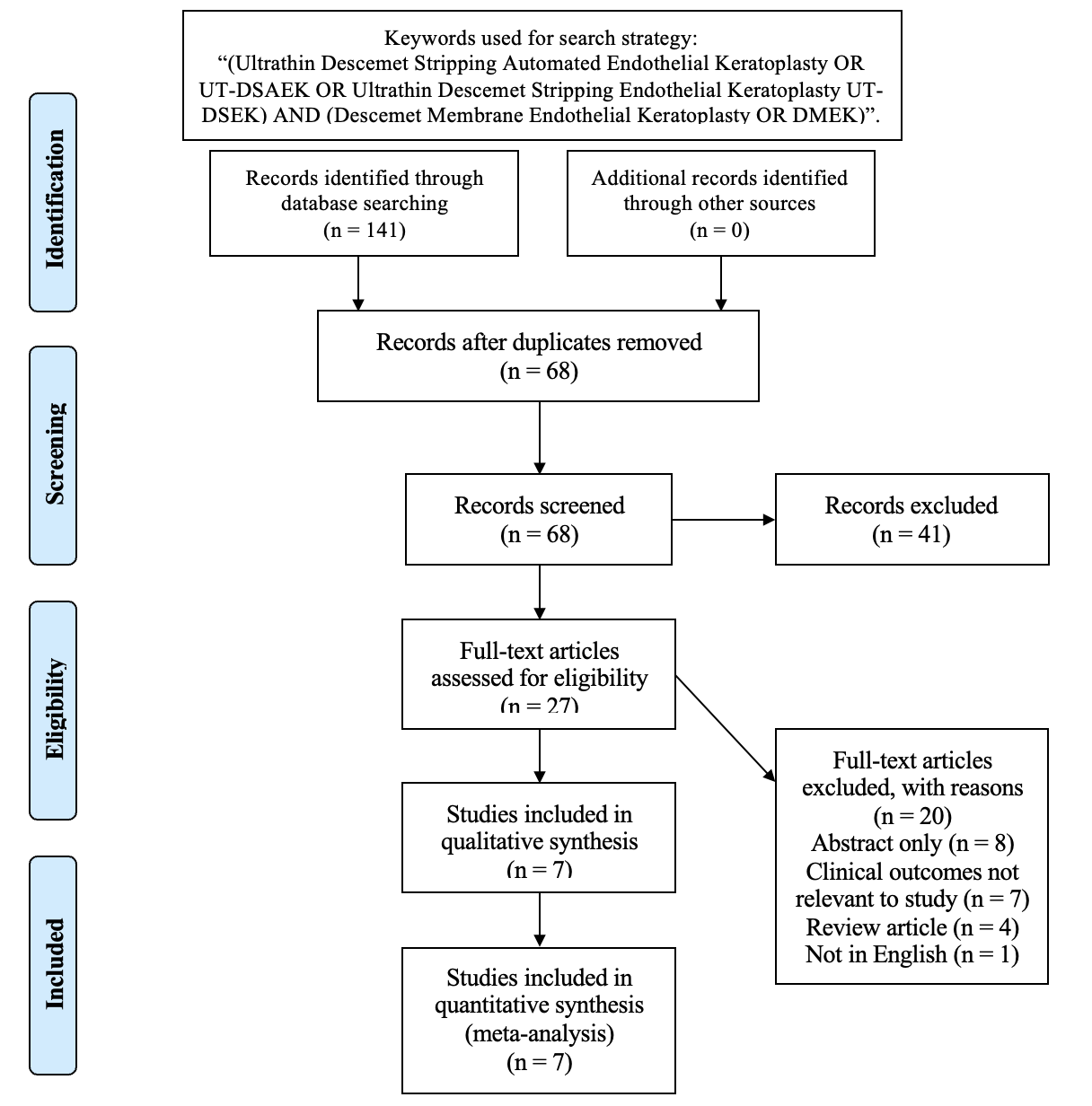
**

| **Appendix 2.** Total Complications | | |
| --- | --- | --- |
| Complication | UT-DSAEK | DMEK |
| Re-Bubbling | 11 | 31 |
| Glaucoma / Raised IOP | 15 | 14 |
| Graft Failure | 1 | 2 |
| Graft Rejection | 2 | 1 |
| Re-Transplantation | 0 | 3 |
| Retinal Tear | 0 | 1 |
| Cystoid Macular Edema | 0 | 1 |
| Posterior Synechiae | 0 | 1 |
| Donor Preparation Failure | 1 | 1 |
| Other | 3 | 4 |

**Appendix 3.** Newcastle-Ottawa Scale (NOS) for assessing methodological quality of evidence (MQOE) in cohort studies

| Study | Selection | | | | Comparability | Outcome | | | NOS score |
| --- | --- | --- | --- | --- | --- | --- | --- | --- | --- |
|  | A | B | C | D | E | F | G | H |  |
| Mencucci, 2020 | * | * | * | * | ** |  | * | * | 8 |
| Romano, 2020 | * | * | * | * |  |  | * | * | 6 |
| Torras-Sanvicens, 2021 | * | * | * | * | * |  | * | * | 7 |
| Tourabaly, 2019 | * | * | * | * | * |  | * | * | 7 |

A, Representativeness of the exposed cohort; B, Selection of the non-exposed cohort; C, Ascertainment of exposure; D, Demonstration that outcome of interest was not present at start of study; E, Comparability of cohorts on the basis of the design or analysis controlled for confounders; F, Assessment of outcome; G, Was follow-up long enough for outcomes to occur; H, Adequacy of follow-up of cohorts. * represents 1 point (max 2 points for E)

**Appendix 4.** Cochrane Collaboration risk of bias tool for assessing MQOE in randomised controlled trials


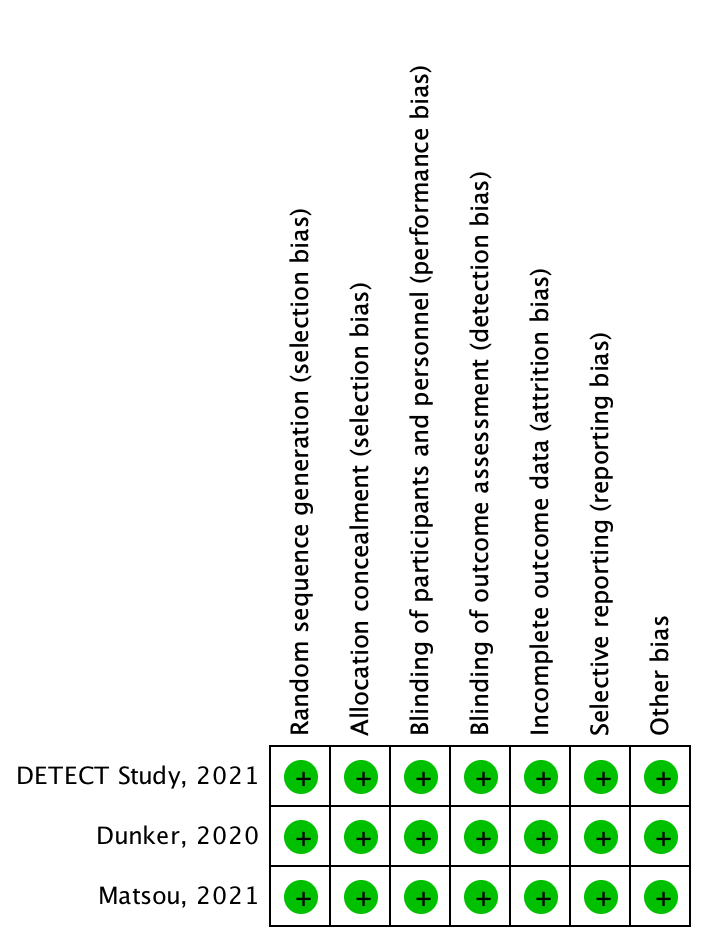


| **UT-DSAEK versus DMEK** | | | | | |
| --- | --- | --- | --- | --- | --- |
| **Patients or population:** Patients with corneal endothelial disease  **Intervention 1:** Ultrathin descemet stripping automated endothelial keratoplasty (UT-DSAEK)  **Intervention 2**: Descemet membrane endothelial keratoplasty (DMEK) | | | | | |
| Outcomes | Illustrative comparative risks | | Relative effect  (95% CI) | No. of eyes  (studies) | Quality of the evidence  (GRADE) |
|  | **UT-DSAEK** | **DMEK** |  |  |  |
| **BCVA** (LogMAR)  *1 year follow-up* | 0.14 | 0.07 | MD: 0.07  (0.04 – 0.10) | 362  (7) | ⊕⊕⊕⊕  high |
| **Endothelial Cell Density** (cells/mm^2^)  *1 year follow-up* | 1,541 | 1,605 | MD: -63.57  (-296.7 – 169.5) | 216  (5) | ⊕⊕⊝⊝  low |
| **Total Complications**  *1 year follow-up* | 33 | 59 | RR: 0.57  (0.36 – 0.9) | 236  (5) | ⊕⊕⊕⊝ moderate |
| **Rates of Re-Bubbling**  *1 year follow-up* | 11 | 31 | RR: 0.40  (0.22 – 0.73) | 236  (5) | ⊕⊕⊕⊕  high |
| The corresponding risk (and its 95% confidence interval) is based on the assumed risk in the comparison group and the relative effect of the intervention (and its 95% CI). CI: Confidence interval; MD: Mean Difference; RR: Risk Ratio | | | | | |
| GRADE Working Group grades of evidence  **High quality:** Further research is very unlikely to change our confidence in the estimate of effect  **Moderate quality:** Further research is likely to have an important impact on our confidence in the estimate of effect and may change the estimate  **Low quality:** Further research is very likely to have an important impact on our confidence in the estimate of effect and is likely to change the estimate  **Very low quality:** We are very uncertain about the estimate. | | | | | |

**Appendix 5.** Summary of Findings
